# Understanding Dry Matter and Nitrogen Accumulation with Time-Course for High-Yielding Wheat Production in China

**DOI:** 10.1371/journal.pone.0068783

**Published:** 2013-07-17

**Authors:** Qingfeng Meng, Shanchao Yue, Xinping Chen, Zhenling Cui, Youliang Ye, Wenqi Ma, Yanan Tong, Fusuo Zhang

**Affiliations:** 1 Center for Resources, Environment and Food Security, China Agricultural University, Beijing, China; 2 College of Resources and Environmental Sciences, Henan Agricultural University, Zhengzhou, China; 3 College of Resources and Environmental Sciences, Hebei Agricultural University, Baoding, China; 4 College of Resources and Environmental Sciences, Northwest Sci-Tech University of Agriculture and Forestry, Yangling, China; China Agricultural University, China

## Abstract

Understanding the time-course of dry matter (DM) and nitrogen (N) accumulation in terms of yield–trait relationships is essential to simultaneously increase grain yield and synchronize N demand and N supply. We collected 413 data points from 11 field experiments to address patterns of DM and N accumulation with time in relation to grain yield and management of winter wheat in China. Detailed growth analysis was conducted at the Zadok growth stages (GS) 25 (regreening), GS30 (stem elongation), GS60 (anthesis), and GS100 (maturity) in all experiments, including DM and N accumulation. Grain yield averaged 7.3 Mg ha^−1^, ranging from 2.1 to 11.2 Mg ha^−1^. The percent N accumulation was consistent prior to DM accumulation, while both DM and N accumulation increased continuously with growing time. Both the highest and fastest DM and N accumulations were observed from stem elongation to the anthesis stage. Significant correlations between grain yield and DM and N accumulation were found at each of the four growth stages, although no positive relationship was observed between grain yield and harvest index or N harvest index. The yield increase from 7–9 Mg ha^−1^ to >9 Mg ha^−1^ was mainly attributed to increased DM and N accumulation from stem elongation to anthesis. Although applying more N fertilizer increased N accumulation during this stage, DM accumulation was not improved, indicating that N fertilizer management and related agronomic management should be intensified synchronously across the wheat growing season to simultaneously achieve high yields and match N demand and N supply.

## Introduction

As the largest wheat producer and consumer in the world, China produced around 115 million t wheat with a planting area of about 24.3 million ha in 2010 [1]. To meet the projected demands of population growth and increasing consumption, cereal production must increase by 70–100% until 2050 [2], [3]. However, as one of major cereal, yield growth of wheat in China has dropped from 9% in 1970s to <3% annually in 2000s [1]. Because of the great difficulty of improving yield potential over the short-term through genetic improvement [4], great efforts should be made to improve agronomical management to enhance average farm yields. Knowledge of DM and nutrient accumulation associated with yield–trait relationships in crops would supply an efficient tool to improve management efficiency and thus increase yield.

Grain yield has usually been positively correlated with total DM production and nutrient accumulation in crops [5]. Meanwhile, DM accumulation and nutrient accumulation vary with growth stage of crops [6]. Many previous studies indicated that DM and N accumulation for wheat occurred mainly pre-anthesis, and that grain yields greatly depended on the translocation of pre-anthesis assimilates and N accumulation [7]–[10]. However, in practice, a significant correlation has also been found between grain yield and DM production post-anthesis in high-yield systems in China [11], [12]. Because few studies have focused on the relationship between grain yield and DM and N accumulation with time-course (e.g., [13]), the pattern of DM and N accumulation pre- and post-anthesis remains unclear.

Due to lack of proper knowledge, most Chinese farmers tried to increase wheat DM and N accumulation as high as possible at earlier stage to pursue high yield in practice. For example, farmers applied a large amount of irrigation and N fertilizer at early stage (*e.g.,* regreening stage, GS25) [14], [15], which leaded to higher DM through more spring tillering. However, higher tiller population would lead to more non-reproductive tillers [16], and more risk of lodging, pests and diseases when it is more than optimum effective plant population. Meanwhile, the non-reproductive tillers would have a negative effect as competitors for assimilates and plant nutrients [17], [18]. Thus, further studies on DM and N accumulation with time-course are needed, which could help to identify the critical stage of optimal spring tillering through management to improve grain yield.

N management also influences the dynamics of DM and N accumulation by crops [19]. For example, N overuse leads to higher plant N concentrations due to so-called “luxury accumulation” by crops, while N shortage decreases plant N concentrations and sacrifices some grain yield [20], [21]. Recently, N recommendations in crop production systems emphasized the need for greater synchrony between crop N demand and the N supply from all sources throughout the growing season [22], [23]. However, most of previous studies have been made to quantify total N accumulation at harvest through yield goals and crop models [20], [24]–[26]. Due to the limited understanding dynamics of N accumulation, N supply in current N management practices often does not match crop demand. For instance, farmers typically apply a large amount of N fertilizer for wheat before sowing (often >50% total N fertilizer), which results in large N losses to the environment because the plant capacity for N accumulation is small during this period [27].

Thus, much effort should be made for dynamic DM and N accumulations associated with yield–trait relationship to improve management efficiency and simultaneously achieve high yields and synchronize N demand and N supply. In this study, we collected 413 field-year data at nine sites in major wheat area in China, to investigate the relationship between DM and N accumulation and grain yield, the pattern of DM and N accumulation for different yield levels with time, and the dynamics of DM and N accumulation with different N management practices.

## Materials and Methods

Eleven field experiments at nine sites in four key winter wheat domains of China were conducted in Dongbeiwang (DBW) in Beijing; Xiangyun (XY), Zhaobao (ZB), and Lankao (LK) in Henan Province; Dingzhou (DZ) and Quzhou (QZ) in Hebei Province; and Yangling (YL) in Shannxi Province. A typical winter wheat–summer maize rotation system was adopted at all sites. No specific permissions were required for these locations. The field studies did not involve endangered or protected species.

The amount and distribution pattern of precipitation varied widely from year to year, as affected by the continental monsoon climate. Wheat was irrigated two to four times based on soil water content at about 100 mm at each time point. More details such as experimental year, soil texture, organic matter content, total N content, Olsen-P, and NH_4_OAc-K in the different experimental locations were shown in Table S1.

### Experimental Design

For the eleven field experiments, the details of treatments, including varieties and N application rates were listed in Table S2. At least three N treatments were included for all eight N level experiments: no N as a control (N-0), an optimal N rate (N-opt), and the farmer’s N practice (FNP). At XY (2008–2009, 2009–2010), LK, DZ and QZ sites, N treatments at sub- or supra-optimal N rates were also applied in experiments with five or six N treatments, including 40%, 50%, 70% and 75% of N-opt, 125%, 130%, and 150% of N-opt according to the experimental design at each site.

The optimal N rate was based on either in-season root zone N management (DBW and QZ sites) or yield goal-based N recommendation (XY, ZB, LK and DZ sites). For the in-season root zone N management (IRNM), wheat growing season was divided into two periods: from sowing to the stem elongation stage, and from the stem elongation stage to the maturity. The amount of N fertilizer applied at the beginning of each growing period was determined by deducting the amount of soil N_min_ (NH_4_^+^–N+NO_3_^−^–N) in the root-zone from the target N value, which was estimated based on the yield target and crop N accumulation. Detailed descriptions of the target N value and the soil N_min_ measurement have been previously reported [27], [28]. The yield goal-based N recommendation was derived from the mass balance approach [29]. In this method, optimal N rate was estimated as total N accumulation of the grain less all of the other sources of grain N and adjusted for inefficiencies in the ability of the crop to recover fertilizer N from the soil [30]:
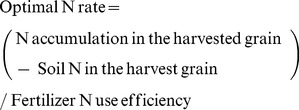
(1)where optimal N rate was estimated for a selected yield goal, fertilizer N use efficiency was the proportion of fertilizer N applied to the soil that was recovered in the grain. For the FNP treatment, the N rate varied from 300 to 360 kg ha^−1^, mostly with a split application (50%∶50%) as a broadcast application at pre-sowing, and as a side-dress application near the stem elongation stage.

For other three cropping systems experiments (DZ, QZ and YL sites), details of the system design were in the Text S1. In this study, the current farmers’ system (FP) and higher yield system from agronomists (HY) were considered N-over systems, while the optimized system (HYHR) and integrated soil–crop management system (ISSM) were considered N-opt systems.

All of the experiments consisted of a randomized complete block design with three to four replications. The plot area varied from 20 m^2^ at XY (2007–2008) to 1800 m^2^ at QZ (2007–2010). Urea as the main N fertilizer has been used for all field experiments. Phosphorus (P), potassium (K) and other nutrients were applied as needed according to soil tests. No organic manure was applied. At all sites, winter wheat was seeded in early to mid-October and harvested the following June. During the study year, no obvious water, weed, pest, or disease stresses were observed during the wheat growing season at any site. No obvious winter killing happened for all field experiments.

### Sampling and Laboratory Procedures

Before sowing, soil samples from the 0–30 cm soil layer were air-dried and sieved to remove un-decomposed plant material. Then the sieved samples were used to measure soil organic matter, total N, Olsen-P, and ammonium acetate-extractable K. To determine DM and N content, plant samples were collected four times: at the regreening (GS25), stem elongation (GS30), anthesis (GS60), and maturity (GS100) stages. Plant samples were dried at 60°C to determine the DM, and sub-samples were taken to measure the N content using the Kjeldahl method. At harvest, areas ranging from 6 m^2^ at XY (2007–2008) to 18 m^2^ at QZ (2007–2010) were harvested manually and dried in an oven at 60°C to calculate grain yield. The grain yield value was adjusted to 14% moisture. The number of grains per square meter was counted manually for at least two rows within 1.0 m, and the number per ear was counted manually to estimate a mean value from more than 20 ears. For the thousand kernel weight (TGW), 1000 grains were randomly counted and weighed.

At XY (2007–2008, 2008–2009, 2009–2010), ZB (2007–2008), LK (2008–2009), QZ (2008–2009), DZ (2008–2010), and YL (2008–2009), the total tillers in the N-opt treatment were investigated before tillering (GS20), before winter (GS23), and at stem elongation, anthesis, and maturity.

### Data Analysis

According to grain yield, all data from the nine sites were divided into three groups: <7, 7–9, and >9 Mg ha^−1^. According this yield range, wheat DM and N accumulation were analyzed with time -course. According N application, all data were also divided into three groups: N-0, N-opt and N-over (125%, 130%, and 150% of N-opt, and FNP) for DM and N accumulation analysis. The data were then further categorized into nine groups according to the yield ranges (1) <7 Mg ha^−1^, N-0; (2) <7 Mg ha^−1^, N-opt; (3) <7 Mg ha^−1^, N-over; (4) 7–9 Mg ha^−1^, N-0; (5) 7–9 Mg ha^−1^, N-opt; (6) 7–9 Mg ha^−1^, N-over; (7) >9 Mg ha^−1^, N-0; (8) >9 Mg ha^−1^, N-opt, (9) >9 Mg ha^−1^, N-over. The investigated stems dynamics in the N-opt treatment at six sites (XY (2007–2008, 2008–2009, 2009–2010), ZB (2007–2008), LK (2008–2009), QZ (2008–2009), DZ (2008–2010), and YL (2008–2009)) were also grouped according to grain yield (<7, 7–9, and >9 Mg ha^−1^).

For all collected data, the correlation coefficients (*r*) between grain yield and average yield components, DM production, and N accumulation at different growth stages, were analyzed. At harvest, the amount of DM production translocation was calculated as the difference between dry DM in the stover at anthesis and at harvest. N accumulation translocation was calculated as the difference between N accumulation in the stover at anthesis and at harvest. The rate of fertile tillers from stem-elongation to anthesis was calculated according the result that the number of total ears at anthesis was divided by the tiller number at stem-elongation stage. N accumulation was calculated as:
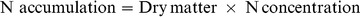
(2)


At regreening, stem elongation and anthesis stage, N accumulation was calculated as straw DM multiplying straw N concentration. At maturity, N accumulation was the sum of straw and grain N accumulation.

## Results

### Grain Yield, DM and N Accumulation, and N Concentration

Overall, wheat grain yield averaged 7.3 Mg ha^−1^, ranging from 2.1 Mg ha^−1^ to 11.2 Mg ha^−1^ (Table 1). According to published statistical data, the average wheat yield was 4.7 Mg ha^−1^ for China and 3.0 Mg ha^−1^ for the whole world in 2009 [1]; thus, the mean yield observed in the present study is 155% and 243% higher than the national and world yields, respectively. However, this is still lower than that in high-yield wheat production areas of Europe, which produce more than 8.0 Mg ha^−1^ [1].

**Table 1:** Descriptive statistics of yield for total samples, three yield ranges, and nitrogen levels (Mg ha^−1^).

	n^a^	Mean	SD^b^	Minimum	25%Q^c^	Median	75%Q	Maximum
Total	413	7.3	2.1	2.1	5.8	7.5	9.2	11.2
Yield category								
<7 Mg ha^−1^	179	5.3	1.3	2.1	4.2	5.6	6.4	7
7–9 Mg ha^−1^	112	8.1	0.6	7	7.5	8.1	8.6	9
>9 Mg ha^−1^	122	9.7	0.5	9	9.4	9.6	9.8	11.2
N-level								
N-0	79	6.6	2.5	2.1	4.1	7.3	8.7	10.4
N-opt	126	7.6	2	3.1	6.1	7.5	9.4	11.2
N-over	154	7.5	1.8	3.1	6.2	7.7	9.3	10.6

a n = number of observation.

b SD = standard deviation.

c Q = quartile.

**Figure 1:**
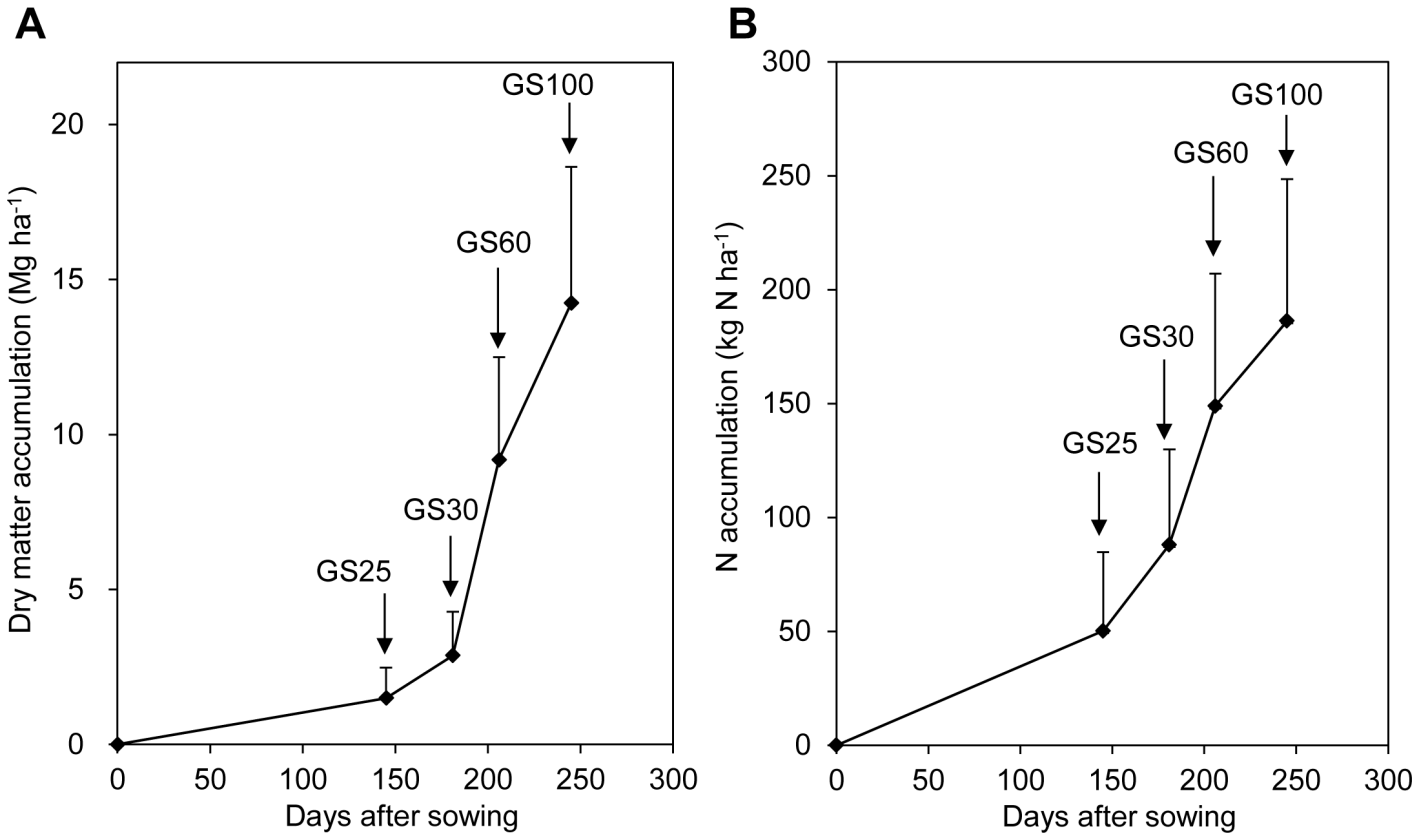
Dynamics of dry matter (A) and nitrogen (B) accumulation from sowing to maturity (n = 413). (GS25, GS30, GS60, and GS100 are the regreening, stem elongation, anthesis, and maturity stages, respectively.).

Sixty-four percent of total DM accumulated pre-anthesis, while the other 36% accumulated post-anthesis (Figure S1). Before anthesis, 10% and 20% of DM accumulated in the regreening and stem-elongation stages, respectively. From stem elongation to anthesis, DM accumulated the most and the fastest across all four stages. More than 6.3 Mg ha^−1^ DM accumulated from stem elongation to anthesis, with an average accumulation rate of 253 kg ha^−1^ day^−1^ (Figure 1A). A total of 1.5 Mg ha^−1^ DM accumulated from sowing to regreening, 1.4 Mg ha^−1^ DM from regreening to stem elongation, and 5.1 Mg ha^−1^ DM accumulated post-anthesis, while the associated daily accumulation rate averaged 21, 38, and 130 kg ha^−1^ day^−1^, respectively.

The percent N accumulation was consistent prior to DM accumulation (Figure S1). Unlike DM accumulation, 80% of N accumulated pre-anthesis; 27%, 20%, and 33% of N accumulated from sowing to regreening, from regreening to stem elongation, and from stem elongation to anthesis, respectively. Similar to DM accumulation, the highest and fastest N accumulation was observed from stem elongation to anthesis. During this period, 2.44 kg N ha^−1^ accumulated per day and 61 kg N ha^−1^ accumulated in total (Figure 1B). Moreover, 50 kg ha^−1^ N, with 0.7 kg N ha^−1^ per day, accumulated from sowing to regreening; 38 kg ha^−1^ N, with 1.1 kg N ha^−1^ per day, accumulated from regreening to stem elongation; and 37 kg ha^−1^ N, with 1.0 kg N ha^−1^ per day, accumulated post-anthesis.

The relationships between grain yield and DM production, N accumulation, and N concentration for different growth stages and yield components were shown in Table 2. For yield components, grains per ear was significantly correlated with grain yield (r = 0.54). Significant correlations between DM and grain yield were observed in each stage (0.58–0.95). Grain yield was significantly correlated with DM accumulation from stem elongation to anthesis and post-anthesis. No positive correlation was found between grain yield and harvest index.

**Table 2:** Average yield components, dry matter and nitrogen accumulation in different growth stages, and correlative coefficient (r) between grain yield and these parameters for all samples (n = 413).

			Mean	r
Yield components	Ears number (10^4^ ha^−1^)		600	0.36
	Grains per ear		39	0.54**
	Grain weight (g 1000^−1^)		41	0.10
Dry matteraccumulation	Regreen (Mg ha^−1^)		1.5	0.60**
	Regreen to stem elongation(Mg ha^−1^)		1.4	0.29
	Stem elongation (Mg ha^−1^)		2.9	0.58**
	Stem elongation to anthesis(Mg ha^−1^)		6.3	0.65**
	Anthesis (Mg ha^−1^)		9.2	0.82**
	Post-anthesis (Mg ha^−1^)		5.1	0.70**
	Maturity (Mg ha^−1^)		14.2	0.95**
	Translocation (Mg ha^−1^)		1.3	0.29
N accumulation	Regreen (kg ha^−1^)		50	0.54**
	Regreen to stem elongation(kg ha^−1^)		38	0.24
	Stem elongation (kg ha^−1^)		88	0.59**
	Stem elongation to anthesis(kg ha^−1^)		61	0.37
	Anthesis (kg ha^−1^)		149	0.71**
	Post-anthesis (kg ha^−1^)		37	0.23
	Maturity (kg ha^−1^)		186	0.81**
	Translocation (kg ha^−1^)		97	0.47**
N concentration	Regreening-straw (%)		3.45	−0.29
	Stem elongation-straw (%)		3.14	0.00
	Anthesis-straw (%)		1.66	−0.14
	Maturity-straw (%)		0.63	0.24
	Maturity-grain (%)		2.15	−0.43**
HI and NHI	Harvest index		0.45	0.02
	N harvest index		0.74	−0.38

** *p*<0.01.

Similar to DM production, significant correlations between grain yield and N accumulation were found at each of stage (r = 0.54–0.81; Table 2). The amount of N translocation from stover to grain was important for yield increase (r = 0.47), although DM translocation was not significant. Between grain yield and N concentration, no significant relationship was found with an exception of grain N concentration at maturity (Table 2). Moreover, no significant correlation was found between grain yield and N harvest index.

### Dynamics of DM and N Accumulation with Different Yield Levels

To further understand the relationship between yield and temporal dynamics of DM and N accumulation, the collected data were further categorized into three groups according to the yield range (Table 1). In total, 179, 112, and 122 data points were grouped according to the yield ranges <7 Mg ha^−1^, 7–9 Mg ha^−1^, and >9 Mg ha^−1^, respectively. The grain yield averaged 5.3 Mg ha^−1^ (2.1–7.0 Mg ha^−1^), 8.1 Mg ha^−1^ (7.0–9.0 Mg ha^−1^), and 9.7 Mg ha^−1^ (9.0–11.2 Mg ha^−1^), respectively. According to the yield components, the yield difference was mainly related to the grain number per square meter (Table S3), which was determined by ear number and grains per ear. The grain number per square meter averaged 24,000 m^−2^ for 7–9 Mg ha^−1^ and 28,000 m^−2^ for >9 Mg ha^−1^, an increase of 33% and 56% compared to the yield range of <7 Mg ha^−1^ (18,000 m^−2^). Similar grain weight per 1000 grains (40–42 g) was observed among all three yield levels.

**Figure 2:**
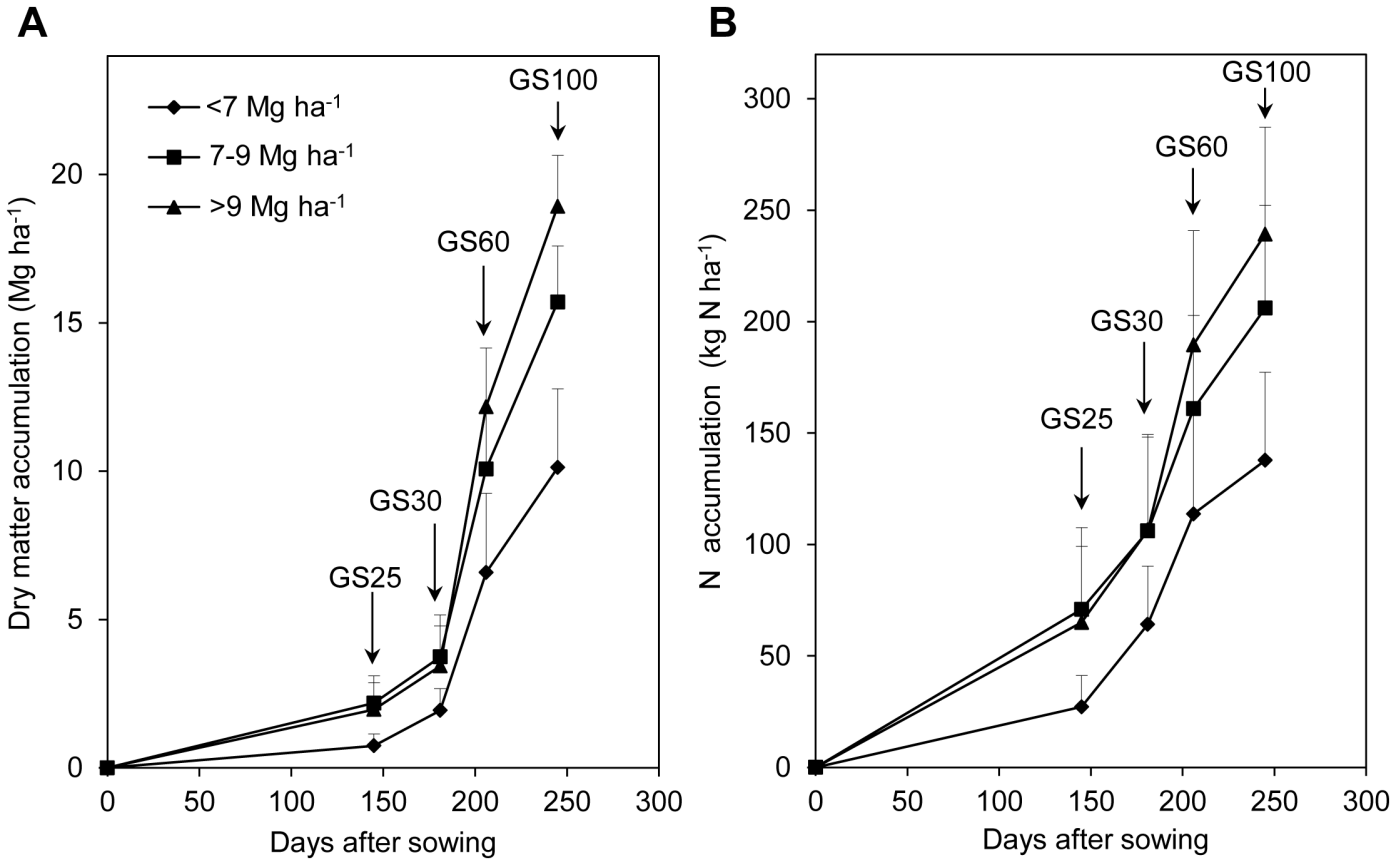
Total dry matter (A) and nitrogen (B) accumulation dynamics at yield ranges of <7 Mg ha^−1^ (n = 179), 7–9 Mg ha^−1^ (n = 112), and >9 Mg ha^−1^ (n = 122). (GS25, GS30, GS60, and GS100 are the regreening, stem elongation, anthesis, and maturity stages, respectively.).

Across the whole wheat season, wheat DM and N accumulation of the yield for <7 Mg ha^−1^ were consistently lower than those for 7–9 Mg ha^−1^ and >9 Mg ha^−1^ (Figure 2). This difference started from the beginning of the growth stage. From the sowing to the regreening stage, 2.2 Mg ha^−1^ DM accumulated in 7–9 Mg ha^−1^, 314% higher than that in <7 Mg ha^−1^ (0.7 Mg ha^−1^) (Figure 2A). Similarly, the 71 kg ha^−1^ of N that accumulated in 7–9 Mg ha^−1^ is substantially higher than the 27 kg ha^−1^ of N that accumulated in <7 Mg ha^−1^ during this stage (Figure 2B). At regreening stage, the percentage of DM (7%) and N accumulation (20%) to total in <7 Mg ha^−1^ was 47% and 43% lower compared with that in 7–9 Mg ha^−1^ yield range (14% and 34%), respectively. The lower DM and N accumulation starting at sowing to regreening and continuing through the subsequent growth stages in yield range <7 Mg ha^−1^, suggested poor agronomic management (e.g., sowing quality, late sowing) may be the main cause, as the yield potential and weather conditions were similar at all sites.

Compared to the yield of 7–9 Mg ha^−1^, DM and N accumulation at a yield of >9 M ha^−1^ was similar before the stem-elongation stage (Figure 2). The greatest difference between these two yield levels for DM and N accumulation took place from stem elongation to anthesis. During this stage, DM accumulation increased from 6.3 Mg ha^−1^ in 7–9 Mg ha^−1^ to 8.7 Mg ha^−1^ in >9 Mg ha^−1^ and was associated with an increase in N accumulation from 55 kg ha^−1^ to 83 kg ha^−1^. Meanwhile, the 46% of DM and 35% of N accumulation to total from stem elongation stage to anthesis in >9 Mg ha^−1^ was 15% and 30% higher compared with that in 7–9 Mg ha^−1^ (40% and 27%), respectively. These results showed that DM and N accumulation from stem elongation to anthesis was very important to achieve the >9 Mg ha^−1^ grain yield. Post-anthesis, 5.6 Mg ha^−1^ DM and 45 kg ha^−1^ N accumulated in 7–9 Mg ha^−1^ while DM and N accumulation averaged 6.8 Mg ha^−1^ and 50 kg ha^−1^ in >9 Mg ha^−1^, respectively.

The yield range <7 Mg ha^−1^ group always had the highest N concentration for all growth stages. At regreening stage, the N concentration in <7 Mg ha^−1^ averaged 3.72%, 13–16% higher than that in both 7–9 Mg ha^−1^ and >9 Mg ha^−1^ yield ranges (3.21–3.29%). At stem elongation stage, the N concentration in <7 Mg ha^−1^ group was 3.32%, 6–16% higher than that in other two yield ranges (2.85–3.13%). At anthesis, 1.75% of N concentration in <7 Mg ha^−1^ was 7–13% higher than in other two yield ranges (1.55–1.63%). At maturity, N concentration in <7 Mg ha^−1^ averaged 1.36%, 4–8% higher than that in other two yield ranges (1.26–1.31%). This may be because of the lower DM accumulation in <7 Mg ha^−1^. For yield ranges 7–9 Mg ha^−1^ and >9 Mg ha^−1^, N concentration was similar for most stages but not at stem elongation stage. At stem elongation stage, N concentration in >9 Mg ha^−1^ was 3.13%, 10% higher than that in 7–9 Mg ha^−1^ (2.85%).

**Figure 3:**
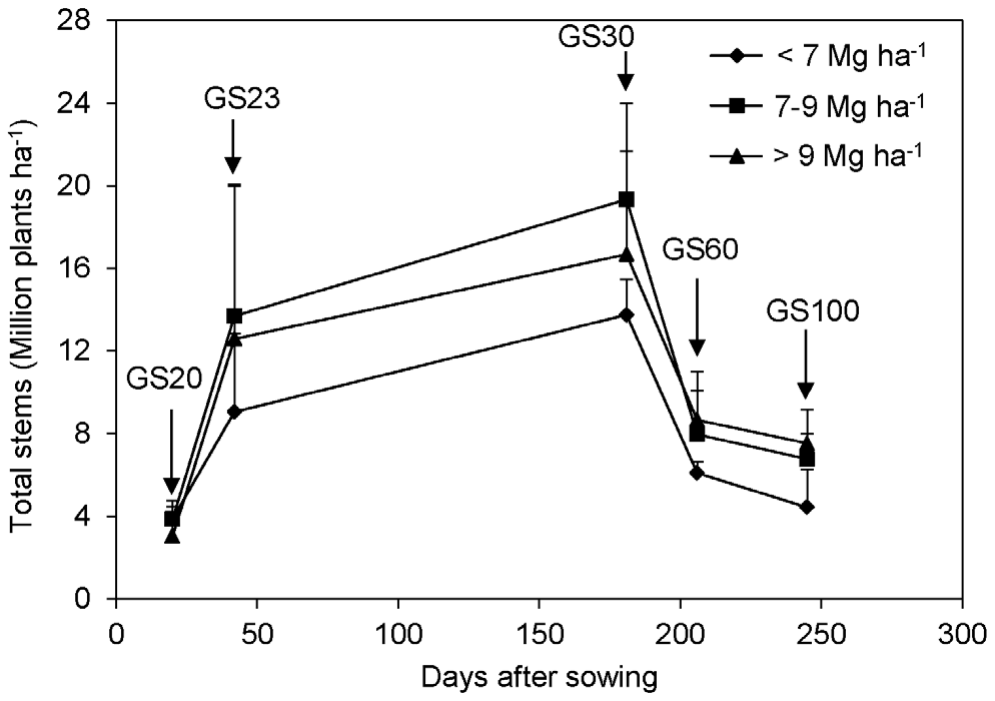
Wheat stem dynamics at grain yields of <7 Mg ha^−1^ (n = 19), 7–9 Mg ha^−1^ (n = 21), and >9 Mg ha^−1^ (n = 33) with optimized N treatment. (GS20, GS23, GS30, GS60, and GS100 are the pre-tillering, before winter, stem elongation, anthesis, and maturity stages, respectively.).

To further understand DM and N accumulation with time in the three yield levels, wheat stem dynamics was investigated at six sites with optimized N treatment (Figure 3). Although the basic seedling was similar among the three yield ranges, total stems differed before winter. Total stems in <7 Mg ha^−1^ was consistently lower compared to that in 7–9 Mg ha^−1^ and >9 Mg ha^−1^ from wintering to maturity. This further verified that sowing quality or other related agronomic management practices were poor and did not supply the optimal growth conditions for wheat. The final ear number was 0.77 million ha^−1^ higher in 9 Mg ha^−1^ than in 7–9 Mg ha^−1^, although total tillers was 2.74 million ha^−1^ higher in 7–9 Mg ha^−1^ in the stem-elongation stage. As a result, earbering tiller percentage averaged 44% (from 16.69 to 7.36 million ha^−1^) in >9 Mg ha^−1^ from the stem-elongation stage to maturity while it averaged only 34% (from 19.43 to 6.59 million ha^−1^) in 7–9 Mg ha^−1^. This suggested that the final ear number was determined by tiller quality, not total number, in earlier growth stages. In the stem-elongation stage, the N concentration in the stover for >9 Mg ha^−1^ averaged 3.27 g kg^−1^, 7% higher than the 3.07 g kg^−1^ for 7–9 Mg ha^−1^ (Table 3). The higher N concentration in the plant in the stem-elongation stage may lead to higher stem quality (e.g., higher tiller weight), and thus more stems would survive from stem elongation to anthesis.

**Table 3:** Stover nitrogen concentration (%) from stem elongation to maturity and grain N concentration at maturity for the N-opt treatment for yield ranges of <7 Mg ha^−1^ (n = 19), 7–9 Mg ha^−1^ (n = 21), and >9 Mg ha^−1^ (n = 33), in which total stem dynamics was investigated.

	Stover			Grain
	Stem elongation (GS30)	Anthesis (GS60)	Maturity (GS100)	Maturity (GS100)
<7 Mg ha^−1^	3.40±0.48^a^	1.74±0.18	0.50±0.07	2.30±0.14
7–9 Mg ha^−1^	3.07±0.45	1.67±0.32	0.58±0.21	2.20±0.20
>9 Mg ha^−1^	3.27±0.54	1.56±0.20	0.62±0.18	1.99±0.31

a average±SD.

### Dynamics of DM and N Accumulation with Different N Management Regimes

**Figure 4:**
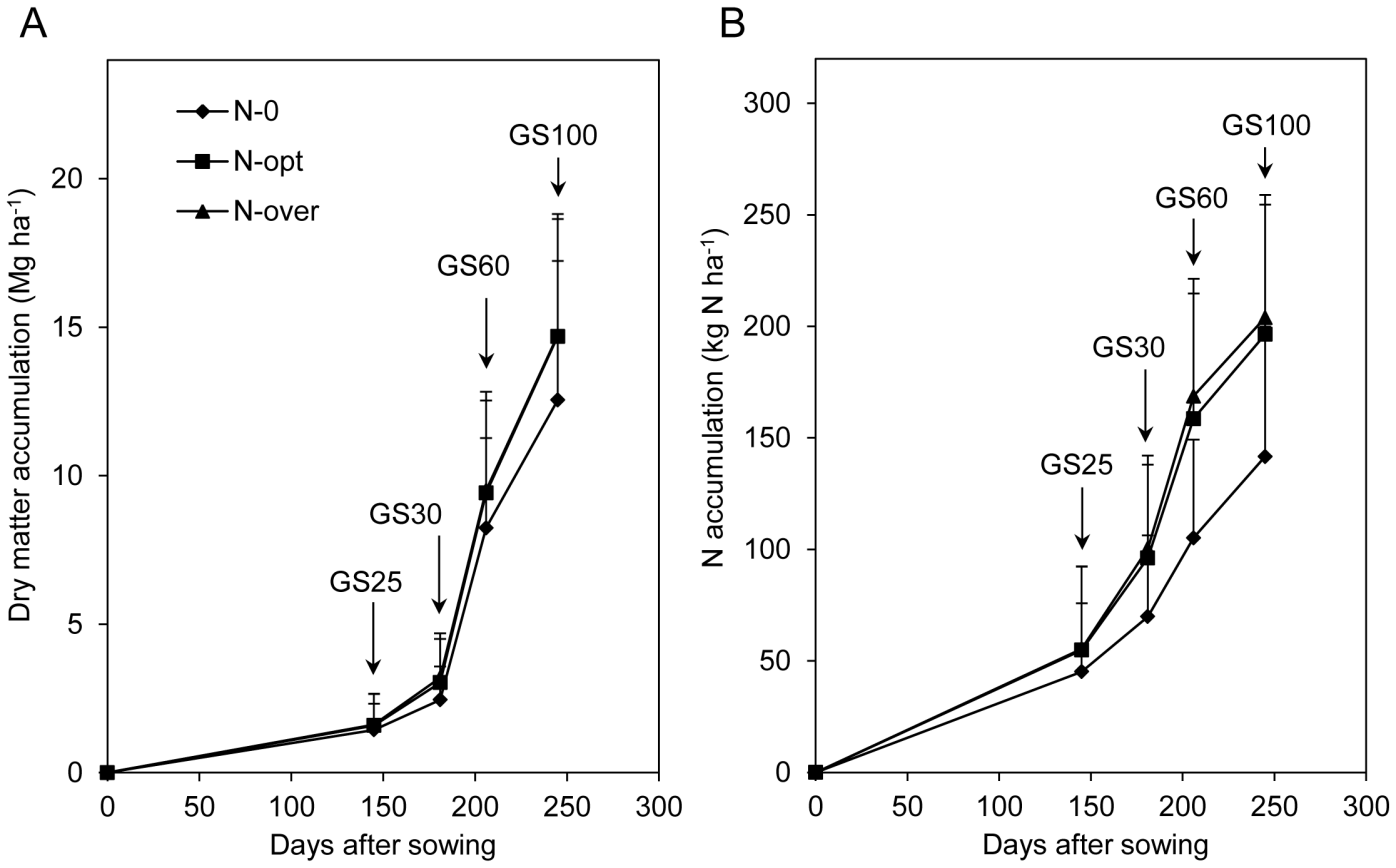
Total dry matter (A) and nitrogen (B) accumulation dynamics with N-0 (n = 79), N-opt (n = 126) and N-over (n = 154) treatments. (GS25, GS30, GS60, and GS100 are the regreening, stem elongation, anthesis, and maturity stages, respectively.).

To further compare the partitioning of DM and N accumulation among different N application and yield levels, pooled data from all nine sites were grouped into three N application categories: N-0, N-opt, and N-over (Table 1). Accordingly, 79 samples with a mean yield of 6.6 Mg ha^−1^ (2.1–10.4 Mg ha^−1^), 126 samples with a mean yield of 7.6 Mg ha^−1^ (3.1–11.2 Mg ha^−1^), and 154 samples with a mean yield of 7.5 Mg ha^−1^ (3.1–10.6 Mg ha^−1^) were grouped according to these three N application levels. Compared to the N-0 treatment, DM and N accumulation increased for the N-opt and N-over treatments in each growth stage for each yield range (Figure 4). Between N-opt and N-over treatments, DM accumulation was similar at each growth stage (Figure 4A). N accumulation was also similar between N-opt and N-over treatments in most growth stages but not at anthesis (Figure 4B). N accumulation averaged around 55 kg ha^−1^ at regreening stage and 100 kg ha^−1^ at stem elongation stage for both N-opt and N-over treatments. At anthesis, N accumulation in N-opt averaged 159 kg ha^−1^, 6% lower compared with that in N-over (169 kg ha^−1^). At maturity, N accumulation averaged about 200 kg ha^−1^ for both N-opt and N-over treatments. However, the N rate in all N-opt management averaged 179 kg ha^−1^ (n = 126), just 59% of the 304 kg ha^−1^ N rate in N-over (n = 154). This showed that more N fertilizer input over the rational level did not lead to more DM and N accumulation except N accumulation from stem elongation to anthesis, and not resulted in a higher grain yield.

**Figure 5:**
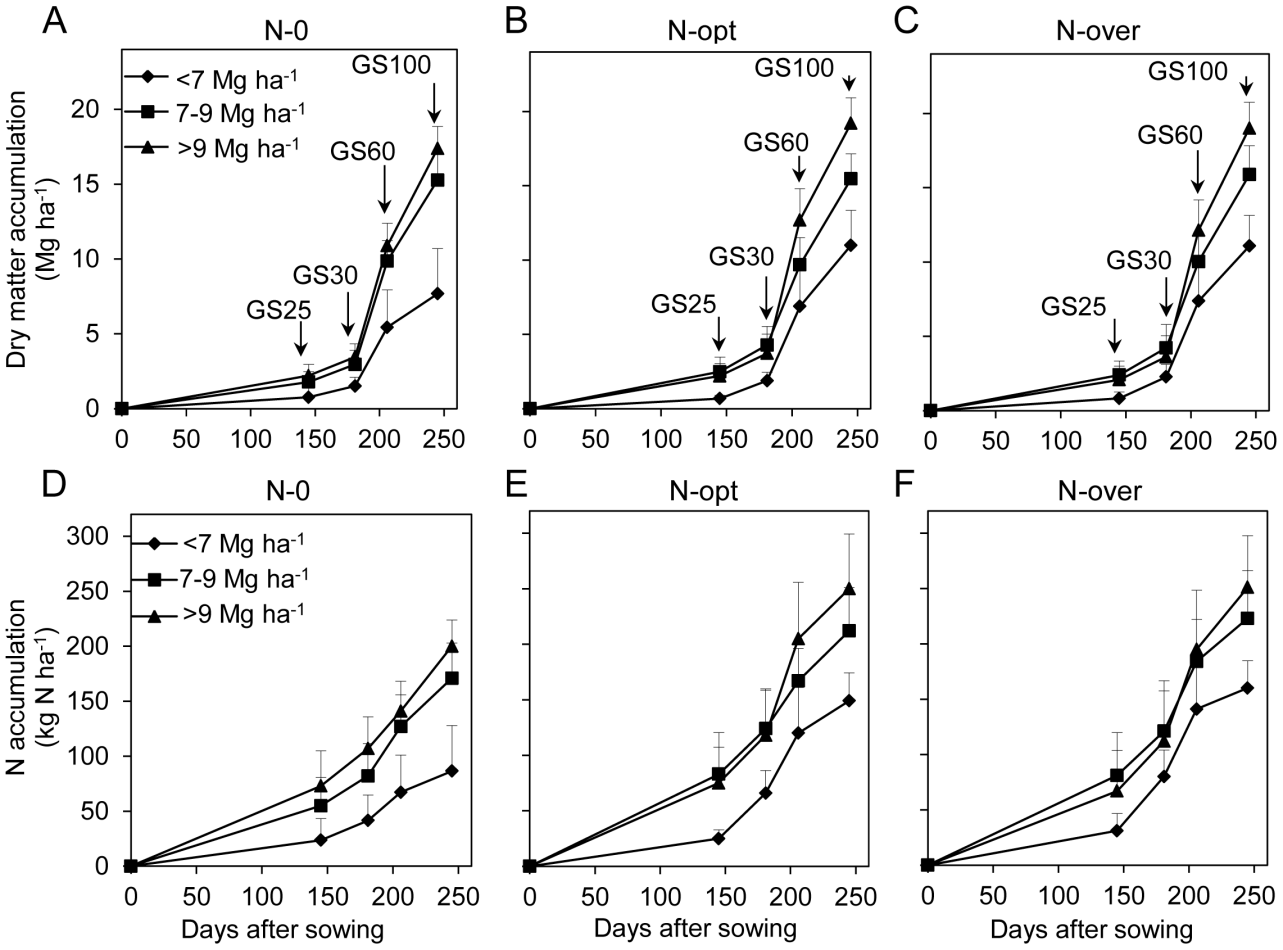
Total dry matter and nitrogen accumulation dynamics in the yield ranges <7, 7–9, and >9 Mg ha^−1^ with N-0 (n = 32, 34, and 13) (A and D), N-opt (n = 56, 29, and 41) (B and E), and N-over treatments (n = 67, 41, and 46) (C and F). (GS25, GS30, GS60, and GS100 are the regreening, stem elongation, anthesis, and maturity stages, respectively.).

For further comparison, the data for each N level were categorized according to the three yield ranges (<7, 7–9, and >9 Mg ha^−1^). DM and N accumulation in the <7 Mg ha^−1^ yield was consistently lower than those in the 7–9 and >9 Mg ha^−1^ (Figure 5). When N was deficient, the difference in N accumulation occurred throughout the whole growing season in the 7–9 and >9 Mg ha^−1^ yield ranges, and the difference in DM accumulation was mainly post-anthesis (Figure 5A, D). Post-anthesis, DM accumulation in >9 Mg ha^−1^ averaged 6.5 Mg ha^−1^ (10.9–17.4 Mg ha^−1^), 20% higher than that in 7–9 Mg ha^−1^ (9.9–15.3 Mg ha^−1^) in the N-0 treatment (Figure 5A). When N was applied rationally, the differences in DM and N accumulation between the yield ranges of 7–9 and >9 Mg ha^−1^ occurred from stem elongation to maturity, particularly during the stem elongation to anthesis stage (Figure 5B, E). During this stage, 9.0 Mg ha^−1^ DM accumulation in >9 Mg ha^−1^ was 67% higher than that in 7–9 Mg ha^−1^ (5.4 Mg ha^−1^), while 40 kg ha^−1^ more N accumulated in >9 Mg ha^−1^. Post-anthesis, 0.7 Mg ha^−1^ more DM accumulated in >9 Mg ha^−1^ than in 7–9 Mg ha^−1^, while the same 45 kg ha^−1^ N accumulated in both yield ranges. Unlike N-opt, the N accumulation was similar between yield ranges of 7–9 and >9 Mg ha^−1^ from sowing to anthesis when N was overused (Figure 5F). At anthesis, N accumulation averaged around 190 kg ha^−1^ for both 7–9 Mg ha^−1^ and >9 Mg ha^−1^, which was similar to the N accumulation in >9 Mg ha^−1^ in the same stage in the N-opt treatment. Moreover, DM accumulation was similar at this stage in >9 Mg ha^−1^ for both N-opt and N-over (Figure 5B, C). However, DM in 7–9 Mg ha^−1^ in this stage was 21% lower than that in >9 Mg ha^−1^, while DM for both of these was around 4.0 Mg ha^−1^ in stem-elongation stage in the N-over treatment (Figure 5C). These results indicated that higher N accumulation did not lead to more DM accumulation synchronously when N fertilizer was overused and thus N luxury accumulation occurred during growth, especially from the stem-elongation stage to anthesis.

## Discussion

We found that wheat yield improvement greatly depended on an increase in DM and N accumulation across the whole growing season. This finding contradicts some previous results, which showed that grain yield was mainly associated with the pre-anthesis assimilate contribution to grain filling and greater DM translocation efficiency [31], [32]. This is because improving the harvest index (by introducing dwarfing genes into new varieties) has traditionally been considered the most important process for increasing the wheat yield potential of modern wheat varieties [33]–[35]. However, the utility of this strategy is limited because of the need to maintain sufficient leaf area and stem dry matter for interception of solar radiation, physical support, and storage of assimilates and N used in grain filling [34]. In the present study, the harvest index averaged 0.45, which was not significantly correlated with grain yield (Table 2). Given the relatively small possibility of further increasing yield by improving the harvest index, the greater yield potential must come from increases in net primary productivity (DM) [36]. The view that increasing DM and N accumulation over the entire growing season is important to achieve high grain yield, is also consistent with recent genetic gains in wheat breeding found in some studies [37], [38].

We found a positive correlation between grain yield and DM accumulation post-anthesis (Table 2), in agreement with other research on modern wheat production in China [11], [12]. Furthermore, yield increase was mainly attributable to DM and N accumulation from stem elongation to anthesis and post-anthesis (Figure 2). The stage from stem elongation to anthesis accumulated the most DM and N, and the accumulation rate was also highest among the four growth seasons (Figure 1). In plant physiology, the period a few weeks before anthesis (from terminal spikelet initiation to anthesis) is of paramount importance in determining the number of fertile florets at anthesis and final grain yield [39], [40].

The final ear number was similar or even higher in 9 Mg ha^−1^ than in 7–9 Mg ha^−1^, although total stems was less in >9 Mg ha^−1^ than in 7–9 Mg ha^−1^ in the stem-elongation stage (Figure 3). This indicates that the higher ear number at maturity is likely attributable to population quality, not tiller number. In the stem-elongation stage, the N concentration in >9 Mg ha^−1^ was significantly higher than that in 7–9 Mg ha^−1^ (Table 3). The higher N concentration in plants the stem-elongation stage might lead to higher stem quality (e.g., higher tiller weight) and thus more stems would survive from stem elongation to anthesis. In practice, this suggests that tiller quality is of more importance than total tiller number.

DM accumulation was fastest (253 kg ha^−1^ day^−1^) from stem elongation to anthesis, and then decreased significantly with time (Figure 1A). This differs from wheat production in most European countries. For example, a study in Germany showed that the DM accumulation rate reached 200 kg ha^−1^ day^−1^ during this stage, and then consistently increased to more than 250 kg ha^−1^ per day in the early grain-filling stage [41]. Meanwhile, the grain-filling stage for wheat is longer in Germany than in China because of cool temperatures during this stage. The dry and hot winds in May and June in China’s wheat production area accelerate wheat maturation [42]. The higher DM accumulation rate during the earlier grain-filling stage and the longer grain-filling stage in Germany would lead to higher wheat yields.

In China, pursuing high grain yield has been the top priority in policy and in practice. The typical N rate applied by winter wheat farmers in the North China plain is around 369 kg N ha^−1^ [15], whereas results from region-wide experiments have demonstrated the optimal N rate to be 128 kg N ha^−1^ [27]. Most Chinese farmers apply large amounts of chemical fertilizer at a uniform rate as an “insurance” against low yields. Affected by the Green Revolution, most farmers still believe that more fertilizer and higher grain yield are synonymous. Our results showed applying more N fertilizer than the rational didn’t result in higher wheat yield (Table 1).

On the other hand, being restricted by old knowledge and habits, farmers often apply large amounts of N fertilizer before sowing or during early growth stages (∼50% of total N application). Our present study clearly demonstrated that this large amount of N fertilization before wheat planting was completely unnecessary, and was likely to move beyond the root zone, particularly under irrigated conditions. In addition, surplus N at early stage would lead to more non-reproductive tillers [16], and more risk of lodging, pests and diseases when it is more than optimum effective plant population. To achieve high yield and N use efficiency at the same time, 60–70% of total N fertilizer application should be applied during the rapid crop-growth period, such as stem elongation stages for wheat.

### Conclusions

A lack of information on DM and N accumulation dynamics with different yield ranges and N management regimes has resulted in N overuse and misuse and various environment problems in China and worldwide. We found significant correlations between grain yield and DM and N accumulation in each growth stage. Both the highest and fastest DM and N accumulations were observed from stem elongation to anthesis. During this stage, increased DM and N accumulation in >9 Mg ha^−1^ yield plots was key for a final higher yield compared with 7–9 Mg ha^−1^. Applying more N could increase the N accumulation during this stage; however, DM accumulation was not further improved and thus final yield was not increased. This suggests that N management alone is insufficient to increase grain yield and that associated agronomic management practices should be intensified. Moreover, we found that higher final ear number at harvest was determined by higher tiller quality (e.g., higher tiller weight) in earlier stages, not by tiller number. Thus, more attention should be paid to increasing wheat tiller quality.

## Supporting Information

Figure S1 Changes in dry matter and nitrogen accumulation expressed as a percentage of the levels at maturity (n = 413). (GS25, GS30, GS60, and GS100 are the regreening, stem elongation, anthesis, and maturity stages, respectively).
(TIF)

Table S1 Location, year, soil texture, and selected chemical properties in the top 30-cm soil layer at nine sites in intensive wheat production areas in China.
(DOC)

Table S2 Location, year, variety and treatment number, treatment and N rate for the eleven field experiments.
(DOC)

Table S3 Ear number, grains per ear, grain number per square meter and grain weight per thousand grains for grain yield ranges of <7 Mg ha^−1^ (n = 179), 7–9 Mg ha^−1^ (n = 112), and >9 Mg ha^−1^ (n = 122).
(DOC)

Text S1 Design of the system experiment.
(DOC)
