# Supplementary figures and images for: Understanding Dry Matter and Nitrogen Accumulation with Time-Course for High-Yielding Wheat Production in China
Source: PLoS One. 2013 Jul 17;8(7):e68783. doi: 10.1371/journal.pone.0068783 (PMC3714303; doi:10.1371/journal.pone.0068783)

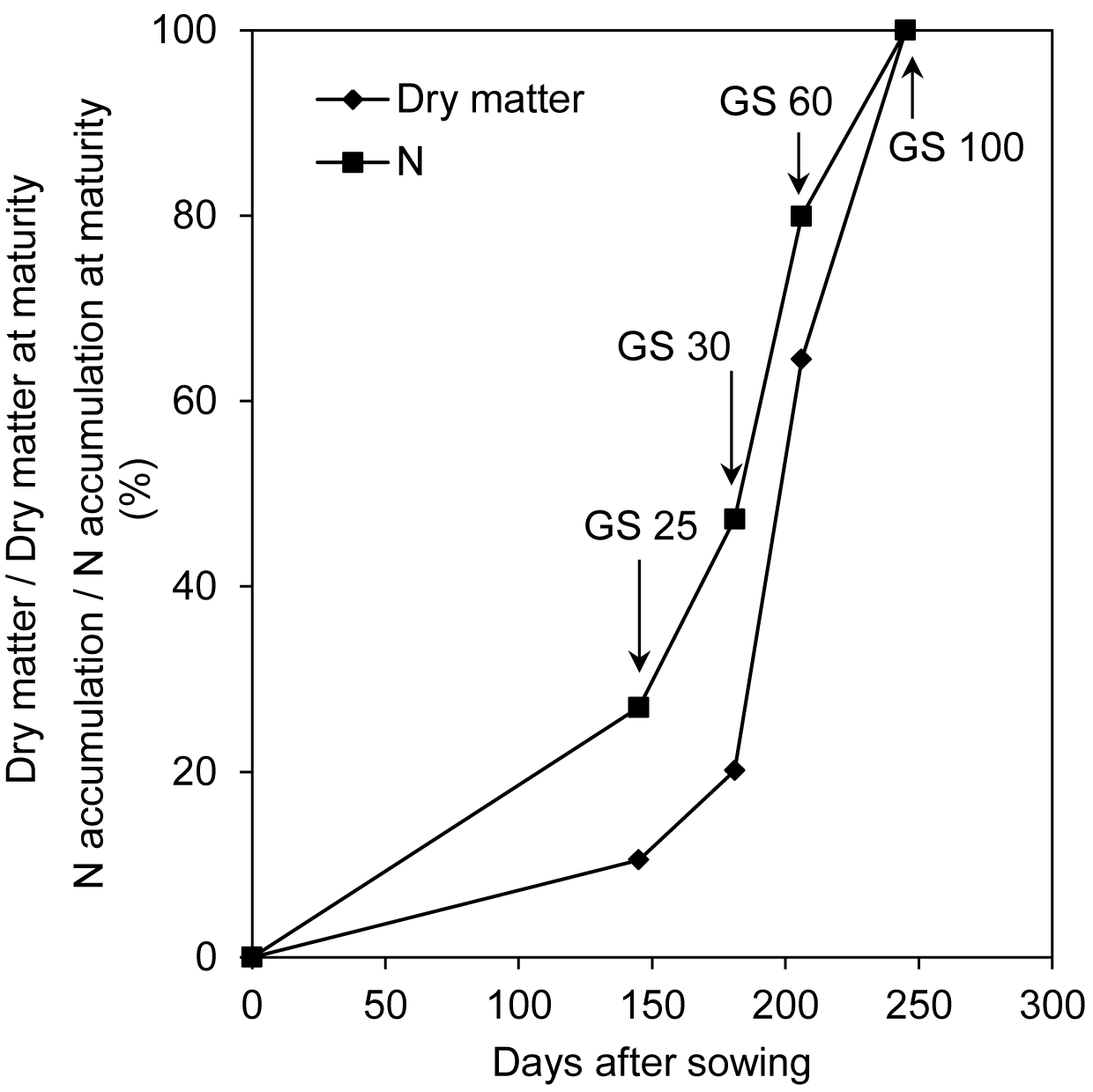

Supplement: Figure S1 — Changes in dry matter and nitrogen accumulation expressed as a percentage of the levels at maturity (n = 413). (GS25, GS30, GS60, and GS100 are the regreening, stem elongation, anthesis, and maturity stages, respectively). (TIF) [file pone.0068783.s001.tif]
