# Supplementary material for: Understanding Dry Matter and Nitrogen Accumulation with Time-Course for High-Yielding Wheat Production in China
Source: PLoS One. 2013 Jul 17;8(7):e68783. doi: 10.1371/journal.pone.0068783 (PMC3714303; doi:10.1371/journal.pone.0068783)
Supplement: Table S1 — Location, year, soil texture, and selected chemical properties in the top 30-cm soil layer at nine sites in intensive wheat production areas in China. (DOC) [file pone.0068783.s002.doc]

**Table S1.** Location, year, soil texture, and selected chemical properties in the top 30-cm soil layer at nine sites in intensive wheat production areas in China.

| **Sites** | **Location** | **Year** | **Soil texture^a^** | **SOM** | **Total N** | **Olsen-P** | **NH_4_OAc-K** |
| --- | --- | --- | --- | --- | --- | --- | --- |
|  |  |  |  | (g kg^-1^) | (g kg^-1^) | (mg kg^-1^) | (mg kg^-1^) |
|  | **Beijing** |  |  |  |  |  |  |
| 1 | DBW | 1999-2000, 2001-2002 | L | 21.4 | 1.17 | 35 | 145 |
|  | **Henan** |  |  |  |  |  |  |
| 2 | XY | 2007-2008 | ML | 10.3 | 1.20 | 20 | 123 |
| 3 | ZB | 2007-2008 | ML | 9.8 | 0.80 | 17 | 105 |
| 4 | XY | 2008-2009 | ML | 15.5 | 1.11 | 26 | 130 |
| 5 | LK | 2008-2009 | ML | 14.5 | 0.92 | 32 | 135 |
| 6 | XY | 2009-2010 | ML | 16.0 | 1.21 | 49 | 153 |
|  | **Hebei** |  |  |  |  |  |  |
| 7 | DZ^b^ | 2008-2010 | L | 16.0 | 0.92 | 12 | 96 |
| 8 | QZ^c^ | 2007-2010 | SL | 12.6 | 0.70 | 5 | 73 |
|  | **Shannxi** |  |  |  |  |  |  |
| 9 | YL | 2008-2009 | CUI | 12.1 | 1.24 | 28 | 147 |

^a^ L for loamy soil, ML for median loam, SL for sandy loam, CUI for Cumuli-Ustic Isohumosols.

^b^ crop system experiment conducted from 2008 to 2010 and the nitrogen-level experiment from 2008–2009.

^c^ Both crop system and nitrogen-level experiments were conducted from 2007 to 2010.
