# Supplementary material for: Understanding Dry Matter and Nitrogen Accumulation with Time-Course for High-Yielding Wheat Production in China
Source: PLoS One. 2013 Jul 17;8(7):e68783. doi: 10.1371/journal.pone.0068783 (PMC3714303; doi:10.1371/journal.pone.0068783)
Supplement: Table S2 — Location, year, variety and treatment number, treatment and N rate for the eleven field experiments. (DOC) [file pone.0068783.s003.doc]

**Table S2.** Location, year, variety and treatment number, treatment and N rate for the eleven field experiments.

|  | **Sites** | **Location** | **Year** | **Variety No.** | **Treatment no.** | **Treatment^a^** |
| --- | --- | --- | --- | --- | --- | --- |
| N level experiment |  | **Beijing** |  |  |  |  |
|  | 1 | DBW | 1999-2000, | 1 | 3 | N-0 (0), N-opt (86), FNP (300) |
|  |  | DBW | 2001-2002 | 1 | 3 | N-0 (0), N-opt (98), FNP (300) |
|  |  | **Henan** |  |  |  |  |
|  | 2 | XY | 2007-2008 | 15 | 3 | N-0 (0), N-opt (150), FNP (300) |
|  | 3 | ZB | 2007-2008 | 15 | 3 | N-0 (0), N-opt (150), FNP (300) |
|  | 4 | XY | 2008-2009 | 2 | 5 | N-0 (0), 50% of N-opt (90), N-opt (180), 150% of N-opt (270), FNP (360) |
|  | 5 | LK | 2008-2009 | 2 | 5 | N-0 (0), 50% of N-opt (90), N-opt (180), 150% of N-opt (270), FNP (360) |
|  | 6 | XY | 2009-2010 | 3 | 6 | N-0 (0), 50% of N-opt (120), 75% of N-opt (180), N-opt (240), 125% of N-opt (300), FNP (360) |
|  |  | **Hebei** |  |  |  |  |
|  | 7 | DZ | 2008-2009 | 1 | 5 | N-0 (0), 40% of N-opt (108), 70% of N-opt (189), N-opt (270), FNP (350) |
|  | 8 | QZ | 2007-2010 | 1 | 5 | N-0 (0), 70% of N-opt (97-139), N-opt (138-198), 130% of N-opt (179-257), FNP (300) |
| Cropping system experiment |  | **Hebei** |  |  |  |  |
|  | 9 | DZ | 2008-2010 | 1 | 4 | FP (270), HYHR (225), HY (300), ISSM (270) |
|  | 10 | QZ | 2007-2010 | 1 | 2 | FP (300), HYHR (100-263) |
|  |  | **Shannxi** |  |  |  |  |
|  | 11 | YL | 2008-2009 | 1 | 4 | FP (300), HYHR (150), HY (270), ISSM (225) |

^a^ The value in parentheses was the N rate.
