# Supplementary material for: Understanding Dry Matter and Nitrogen Accumulation with Time-Course for High-Yielding Wheat Production in China
Source: PLoS One. 2013 Jul 17;8(7):e68783. doi: 10.1371/journal.pone.0068783 (PMC3714303; doi:10.1371/journal.pone.0068783)
Supplement: Table S3 — Ear number, grains per ear, grain number per square meter and grain weight per thousand grains for grain yield ranges of <7 Mg ha−1 (n = 179), 7–9 Mg ha−1 (n = 112), and >9 Mg ha−1 (n = 122). (DOC) [file pone.0068783.s004.doc]

**Table S3.** Ear number, grains per ear, grain number per square meter and grain weight per thousand grains for grain yield ranges of <7 Mg ha^-1^ (n=179), 7–9 Mg ha^-1^ (n= 112), and >9 Mg ha^-1^ (n=122).

|  | **Ear number** (10^4^ ha^-1^) | **Grains per ear** | **Grain number per square meter** (10^4^ m^-2^) | **Grain weight** (g 1000^-1^) |
| --- | --- | --- | --- | --- |
| <7 Mg ha^-1^ | 572±135^a^ | 32±7 | 1.8±0.5 | 40±4 |
| 7-9 Mg ha^-1^ | 592±120 | 42±10 | 2.4±0.5 | 41±5 |
| >9 Mg ha^-1^ | 634±120 | 44±7 | 2.8±0.6 | 42±5 |

^a^ average±SD
