# Supplementary material for: Understanding Dry Matter and Nitrogen Accumulation with Time-Course for High-Yielding Wheat Production in China
Source: PLoS One. 2013 Jul 17;8(7):e68783. doi: 10.1371/journal.pone.0068783 (PMC3714303; doi:10.1371/journal.pone.0068783)
Supplement: Text S1 — Design of the system experiment. (DOC) [file pone.0068783.s005.doc]

**Design of the system experiment**

The cropping system experiment included four treatments: current farmers’ system (FP), optimized system (HYHR), higher yield system from agronomists (HY), and an integrated soil–crop management system (ISSM). The FP system simulated the farmers practice in local production when the HYHR system had the goal of both grain yield and resource use efficiencies increasing 10-15% compared with farmers. For the HY system from agronomists, grain yield was designed to nearly attain the local yield ceiling thoroughly with extensive inputs, regardless of the costs and environmental risks. For the ISSM system, the goal of both grain yield and resource use efficiencies was 30-50% of increase compared with farmers.
